# Integration of immigrants into a new culture is related to poor sleep quality

**DOI:** 10.1186/1477-7525-6-61

**Published:** 2008-08-10

**Authors:** Ursula Voss, Inka Tuin

**Affiliations:** 1 Dept. of Psychology at the J.W. Goethe-University Frankfurt/M., Mertonstr. 17, 60054, Frankfurt/M., Germany; 2 Dept. of Cognitive Psychology at the Rheinische Friedrich-Wilhelms-University Bonn, Kaiser-Karl-Ring 9, 53111, Bonn, Germany; 3 Clinic for Psychosomatic Medicine and Psychotherapy at Johannes Gutenberg University Mainz, Germany

## Abstract

**Background:**

This article reports on the relationship between cultural influences on life style, coping style, and sleep in a sample of female Portuguese immigrants living in Germany. Sleep quality is known to be poorer in women than in men, yet little is known about mediating psychological and sociological variables such as stress and coping with stressful life circumstances. Migration constitutes a particularly difficult life circumstance for women if it involves differing role conceptions in the country of origin and the emigrant country.

**Methods:**

The study investigated sleep quality, coping styles and level of integration in a sample of Portuguese (N = 48) and Moroccan (N = 64) immigrant women who took part in a structured personal interview.

**Results:**

Sleep quality was poor in 54% of Portuguese and 39% of Moroccan women, which strongly exceeds reports of sleep complaints in epidemiologic studies of sleep quality in German women. Reports of poor sleep were associated with the degree of adoption of a German life style. Women who had integrated more into German society slept worse than less integrated women in both samples, suggesting that non-integration serves a protective function. An unusually large proportion of women preferred an information-seeking (monitoring) coping style and adaptive coping. Poor sleep was related to high monitoring in the Portuguese but not the Moroccan sample.

**Conclusion:**

Sleep quality appears to be severely affected in women with a migration background. Our data suggest that non-integration may be less stressful than integration. This result points to possible benefits of non-integration. The high preference for an information-seeking coping style may be related to the process of migration, representing the attempt at regaining control over an uncontrollable and stressful life situation.

## Background

The present study was part of a project investigating the impact of social background on sleep quality in women in Germany. We report on the relationship between cultural influences on life style, coping style, and sleep in a sample of female Portuguese immigrants and compare it to a sample of female Moroccan immigrants living in Germany.

Epidemiological studies conducted in Western societies show poorer sleep quality and a higher proportion of psycho-physiologic sleep disorders such as primary insomnia in women compared to men [1,2]. The incidence of psycho-physiologic sleep disorders is known to be causally related to socio-economic and stress-related psychological variables [for a review see [3]]. Yet, the impact of socio-economic and psychological factors on sleep in relation to gender differences has received little attention.

A recent study found poor sleep quality suggestive of insomnia in 39% of female Moroccan immigrants living in Germany [3], confirming previous findings marking immigration a health and sleep-relevant stress factor [4-9]. By comparison, the prevalence of insomnia in the German population has been found to be relatively low (6.2%) [10]. Surprisingly, Moroccan women who had identified with a more Western lifestyle had a higher incidence of insomnia than women who had retained their traditional Moroccan life style, suggesting that for Moroccan immigrant women in Germany, adopting a Western life style may be more stressful than retaining the native life style. Possibly, non-integration serves a protective function. Further, an unusually large proportion of women preferred an information seeking (monitoring) coping style, which has been shown to be predictive of psycho-physiologic or primary insomnia in Western women [11]. Monitoring is defined by an overreaching desire to obtain information in uncontrollable situations [12], most likely motivated by the attempt to obtain control over a threatening situation [13]. Monitoring constitutes an extreme coping style, which is, however, preferred by as much as 13% of the Western population [14]. Gender differences have been established in German [15], Austrian [14], and Dutch [16], but not in Spanish [17] or North-American samples [12], suggesting that cultural factors play a role. In German, Austrian and Dutch samples women are stronger information seekers than men.

Unlike in Western women [11], elevated monitoring scores in the Moroccan sample were not predictive of poor sleep. Another unusual finding concerns adaptive coping which was also very pronounced in female Moroccan immigrants. Adaptive coping constitutes a flexible approach to handle stress: information is preferred in situations that are controllable through early intervention. By contrast, distraction and avoidance (blunting) are practiced in situations that are not to be controlled. Although adaptive coping constitutes the healthiest stress response, this strategy is only applied by 5% of Western women. By contrast, 19% of the studied Moroccan immigrant women engaged in adaptive coping [14,16]. While these findings suggest that poor sleep and coping style preference are influenced by cultural factors, the observed relationships between life style, coping style, and sleep were not anticipated. To investigate whether these results are typical for female immigrants in Germany, or whether culture-specific factors such as religious preferences or social position of women in their families, for example, have to receive higher weighting, the interview was repeated with a group of female Portuguese immigrants living in Germany, assessing sleep quality, coping style and life style. Women in the Portuguese sample are similar to the Moroccan sample regarding socio-economic factors such as education, age, and immigration status. They differ in their religious orientation (Catholic vs. Moslem) and their stronger proximity to a Western life style in their country of origin [18].

## Methods

### Participants

#### Portuguese sample

The study was approved by the local ethics committee of the Department of Medicine of the J. W. Goethe-University Frankfurt, Germany. Written informed consent was obtained from all participants prior to the interview.

The sample size was planned at 80 participants, assuming a strong effect size of .8 (α = .05, power = 80%) [19]. However, only 48 women agreed to participate. Similar to women in the Moroccan sample, Portuguese women were very hesitant to participate. Several a priori conversations with husbands and other family members were necessary to dispel doubts about the protection of privacy and the purely scientific nature of the investigation. Due to low levels of literacy and poor German language skills, a female Portuguese translator was present at all times. For the same reasons, interviews were conducted individually, each interview lasting for three hours, on average.

Exclusion factors were pregnancy, previously diagnosed sleep disorder, and chronic disease that may be related to sleep quality. Participants were recruited through personal contacts and by word-of-mouth recommendation. Because most women socialized only within small circles of neighbors and family, all participants were residents of the Mainz and Frankfurt urban area. Interviews were conducted at the women's homes or in public places chosen by the participants (coffeehouses, neighborhood circles). Interview questions were translated into Portuguese by professional translators and translated back into German by the accompanying translators. The interview was identical to the one given to the Moroccan women. Internal consistency (Cronbach's alpha) for the PSQI total score was .77 (comparable Moroccan sample: .80 [3]; German sample: .89 [20]; American sample: .82 [21]. The four scales of the FMBS had internal consistencies between .68 (monitoring in controllable situations) and .75 (blunting in uncontrollable situations) (comparable German sample: .70 – .79 [14]. Participants received 20 Euro as compensation for their time.

#### Moroccan sample

A total of 64 Moroccan women participated in the interview. Similar to the Portuguese study, a translator was present for all interviews. Interview questions were identical in both samples and professional translations of structured questions had been translated back into German by the respective translators. A detailed description of the Moroccan sample can be found elsewhere [3]. On average, Moroccan women were younger (31.72 years) than Portuguese women (31.72 vs. 38.60 years, *t *= 2.95, *p *< .01). Portuguese women had been living in Germany longer than Moroccan women (20.29 vs. 14.00 years, *t *= 3.37, *p *< .01).

### Measures

#### Structured Interview

Socio-demographic information, including age at immigration, number of years spent in Germany, years of schooling, and occupational status, were collected in structured interviews. In addition, women were asked to rate their religious customs and life-style-related behaviors. A 4-point rating scale was used in all questions. In accordance to literature reports on influences on cultural identity [22], women were categorized into 4 groups on the basis of four measures: 1) religious customs (self rating of Christian religiousness), 2) self-rated life style (German, partially adapted German, predominantly Portuguese, strictly Portuguese), 3) years since immigration (second generation immigrants, immigration in childhood, less than 10 years or more than 10 years since immigration), 4) translator-rated German language skills (perfectly fluent in German, rather fluent, simple language skills, spoke no German). Language skill ratings were discussed with and in agreement with the participating women. In these four categories, each score received an equal weighting of 25%. Weighted scores were then summed up (score range: 34 – 100) and split into 4 groups: 1) German life-style (scores 25 – 40), 2) rather German life style (scores 41 – 55), 3) rather traditional life style (scores 56 – 70), 4) strictly traditional life style (scores 71 – 85). This procedure matched the one applied to the data of the Moroccan sample. Socio-demographic data for Portuguese and Moroccan women are listed in Table 1 [see additional file 1].

#### Pittsburg Sleep Quality Inventory (PSQI)

Sleep quality was assessed with the Pittsburgh Sleep Quality Index (PSQI), a self-rated questionnaire that provides an index of sleep quality for a 1-month interval [23]. The PSQI comprises 19 questions which are grouped into 7 component scores, each weighted equally on a 0 to 3 scale. The 7 component scores are then totalled to provide a global PSQI score, which has a range of 0 to 21, with higher scores indicating worse sleep quality. The PSQI has established reliability (.82 – .89) and validity (specificity rates to the clinical diagnosis of insomnia: 80 – 100%). Global scores > 6 were interpreted as an indicator of poor sleep quality and a strong indication for insomnia.

#### Frankfurt Monitoring and Blunting Scales (FMBS)

Coping style was assessed by a modified version of the Frankfurt Monitoring Blunting Scales (FMBS). The FMBS have good reliability and validity [14] and comprise four uncontrollable and threatening situations and four controllable yet stressful situations. Each situation is followed by eight behavioral choices, four pertaining to a monitoring and four to a blunting, i.e. distracting style of coping. Participants are asked to anticipate each scenario and rate how likely they would engage in each of the eight behavioral choices. The item scores are summed within each situational type, resulting in four composite scores, one for each monitoring and blunting in uncontrollable and controllable situations. Individuals are classified as rigid "monitors" (high monitoring scores in controllable and uncontrollable situations) or "blunters" (high blunting scores in controllable and uncontrollable situations) or "adaptive copers" (high monitoring scores in controllable situations and high blunting scores in uncontrollable situations) or "unspecified types" on the basis of their scores. Unspecified types refer to participants who are neither monitors nor blunters nor adaptive copers. As in the Moroccan interview, two controllable situations of the original FMBS were exchanged for more housebound situations, because preliminary interviews suggested that the original situations could not be anticipated for cultural reasons. Alterations to the FMBS were identical for both the Moroccan and the Portuguese group.

## Results

As can be seen from table 1 [see additional file 1], 38% of Portuguese women had adopted a German or rather German life style. By contrast, 62% adhere to a life style typical for their country of origin. This result compares well to the data from the Moroccan sample (45% vs. 55%). Most women in both samples were married (79% of Portuguese and 59% of Moroccan women), only one Portuguese woman was divorced (2%) (Moroccan women: 13%). With respect to continuous variables age, years since living in Germany, and years of schooling, analysis of variance (ANOVA) showed that life style was significantly influenced by years of schooling in both samples (Portuguese women: *F *= 15.18, *df *= 3, 44, *p *< .01, Moroccan women: *F *= 5.13 *df *= 3,60, *p *< .01). Posthoc procedures (Scheffé) revealed that a stronger integration (German or rather German life style) was associated with longer schooling. In the Portuguese sample, life style was also influenced by age (*F *= 7.81, *df *= 3, 44, *p *< .01), showing that more integrated women were younger than more traditional women. In the Moroccan sample, integration was favored in women who had lived in Germany longer (*F *= 12.14, *df *= 3,60, *p *< .01).

Regarding coping styles [additional file 1], the percentage of monitors in this sample matched the percentage found in the Moroccan sample (48%). The percentage of adaptive copers was higher than in German (6%) and Austrian women (3%) but lower than in the Moroccan immigrant sample (19%). Similar to the findings in the Moroccan sample, the percentage of blunters in the current sample was inconspicuous.

With respect to sleep, a total of 54% of Portuguese and 39% of Moroccan women had PSQI scores above 6, which is indicative of a sleep disorder. Nonparametric χ^2 ^analyses were performed on PSQI groups (good and poor sleepers) for life style [see additional file 1] and coping style types (monitors, blunters, adaptive copers, unspecified types) [see additional file 2]. Results show significant differences between good and poor sleepers for life style in both groups (Portuguese sample: χ^2 ^= 8.88, *df *= 3, *p *< .05, Moroccan sample: χ^2 ^= 8.11, *df *= 3, *p *< .05). Poor sleep was related to coping style in the Portuguese (χ^2 ^= 8.71, *df *= 3, *p *< .05) but not the Moroccan sample. As can be seen from table 1 [additional file 1], a German life style is associated with poorer sleep than more traditional life styles. Table 2 [additional file 2] shows that the coping style "monitor" is associated with poor sleep in Portuguese women, confirming earlier results reported for German women [11] and standing in contrast with results from the Moroccan immigrant sample which showed better sleep in monitors. Since sleep quality may also be influenced by body mass index (BMI) and age, ANOVAs were conducted on PSQI groups and dependent variables *age *and *BMI*. Both variables did not show significant effects on sleep quality [additional file 2] in either group.

## Discussion

In the sample of Portuguese immigrant women living in Germany, we found similarities with the Moroccan group of female immigrants with regard to sleep quality and life style and dissimilarities with respect to coping.

In both studies, the total percentage of women suffering from poor sleep was higher than that reported in most epidemiologic studies of women in Western culture [24,25], confirming that immigration is a health-relevant life stressor. Further, the fact that the adoption of a German life style was associated with poor sleep in both samples, suggests that the process of societal integration appears to have a negative effect on sleep quality. This confirms the hypothesis that non-integration serves a protective function with respect to sleep quality.

Age and BMI did not significantly affect sleep quality in either sample, although psycho-physiological insomnia has often [e.g. [26]] but not always [27] been found to be elevated around middle age. We can only speculate whether this finding is related to cultural or nutritional factors. It is also possible that the generally more active hypothalamic-pituitary-adrenal axis in women contributes to a greater variability in sleep quality among women of all ages, blurring the specific effects of menopause in this sample [28], for a detailed discussion see [3].

Regarding coping style, the majority of monitors in the Portuguese sample were poor sleepers, while all blunters slept well. This is in accordance with previous findings in German women [11] but in contradiction to the results from the Moroccan sample. Since copings style is independent of age and socio-economic factors, we can only speculate that perhaps the Moroccan women interviewed expressed the wish for information rather than actual coping style. It may also be that anxiety was a moderating factor in this sample. Future studies should, therefore, assess social desirability and anxiety. The overall percentage of monitors in the Portuguese sample (48%) was just as elevated as in the Moroccan sample (48%). In the Portuguese but not the Moroccan sample, monitoring was more frequent in women who had adopted a more German life style. Also, a higher percentage of immigrant women than German or Austrian women preferred adaptive coping, supporting the assumption that coping style is somehow related to the act of immigration. Either a selection bias applies, meaning that women who are monitors or adaptive copers immigrate more readily to a foreign country than women who prefer a non-monitoring coping style. Alternatively, the change of domicile and exposure to a new cultural environment may evoke the need for information as a means of orientation. This would imply that coping style is not so much a personality variable (trait) but modifiable, at least in chronically stressful situations such as immigrating to a foreign country. Further research is needed to establish whether the results found for female German immigrants also apply for immigrants to other countries. Regarding coping style, results generate new hypotheses about the weighting of personality and situative influences on coping with stress.

## Conclusion

The results of this study confirm the finding from the Moroccan immigrant study, showing that integration into German culture is related to poor sleep quality. Moreover, information seeking or monitoring seems to be the most preferred coping style among female immigrants, independent of religious customs and beliefs. Monitoring is more than twice as prevalent in both groups of female immigrants compared to non-immigrant Germans, suggesting that coping style is related to the act of immigration. Uncertainty and insecurity related to life circumstances in a new country may trigger the need to be alert and to excessively seek information, even in uncontrollable situations in which information is not useful. Why poor sleep quality was related to monitoring in the Portuguese sample but not in the Moroccan sample, remains unclear. Possibly, Moroccan women adhering to a traditional Moroccan life style felt a high need for information and expressed their wish for information instead of actual access to it. The finding in the Moroccan sample may also be related to sociological issues like social networking, favouring information exchange between traditional women. Further studies should address these issues.

## Competing interests

The authors declare that they have no competing interests.

## Authors' contributions

Ursula Voss designed the study, instructed and supervised the interviewers, carried out the statistical analyses and drafted the manuscript. Inka Tuin conceived of the study, participated in its coordination and the statistical analysis, and helped to draft the manuscript. All authors read and approved the final manuscript.

## Supplementary Material

Additional file 1 Socio-demographic data. The data provided represent the socio-demographic statistics for the two immigrant samples described. Click here for file

Additional file 2 Descriptive statistics on coping style and sleep quality. This table shows the statistics for coping style and sleep quality in the Portuguese and Moroccan women interviewed. Click here for file
