# Supplementary material for: Integration of immigrants into a new culture is related to poor sleep quality
Source: Health Qual Life Outcomes. 2008 Aug 10;6:61. doi: 10.1186/1477-7525-6-61 (PMC2518135; doi:10.1186/1477-7525-6-61)
Supplement: Additional file 1 — Socio-demographic data. The data provided represent the socio-demographic statistics for the two immigrant samples described. [file 1477-7525-6-61-S1.doc]

Table 1

Socio-demographic data for a sample of Portuguese (N = 48) and Moroccan immigrant women (N = 64)

|  | ***German life style*** | | ***rather German life style*** | | ***rather traditional life style*** | | ***traditional life style*** | | *total* | |
| --- | --- | --- | --- | --- | --- | --- | --- | --- | --- | --- |
|  | *Portuguese*  *f (%)* | *Moroccan*  *f (%)* | *Portuguese*  *f (%)* | *Moroccan*  *f (%)* | *Portuguese*  *f (%)* | *Moroccan*  *f (%)* | *Portuguese*  *f (%)* | *Moroccan*  *f (%)* | *Portuguese*  *f (%)* | *Moroccan*  *f (%)* |
| **Marital status**  single  married  divorced  widowed | 3 (50%)  3 (50%)  0 ( 0%)  0 ( 0%) | 4 (57%)  2 (29%)  1 (24%)  0 ( 0%) | 4 (33%)  7 (58%)  1 ( 9%)  0 | 8 (36%)  9 (41%)  5 (23%)  0 ( 0%) | 0 ( 0%)  15 (100%)  0 ( 0%)  1 | 3 (25%)  9 (75%)  0 ( 0%)  0 ( 0%) | 1 ( 7%)  13 (93%)  0 ( 0%)  0 ( 0%) | 3 (13%)  18 (78%)  2 ( 9%)  0 ( 0%) | 8 (17%)  38 (79%)  1 ( 2%)  1 ( 2%) | 18 (28%)  38 (59%)  8 (13%)  0 (0%) |
| total | 6 (13%) | 7 (11%) | 12 (25%) | 22 (34%) | 16 (33%) | 12 (19%) | 14 (29%) | 23 (36%) | 48 (100%) | 64 (100%) |
| **Coping style ^1)^**  Monitors  Blunters  Adaptive C  unspecified | 2 (33%)  2 (33%)  0 ( 0%)  2 (33%) | 4 (57%)  1 (14%)  2 (29%)  0 ( 0%) | 8 (67%)  2 (17%)  1 ( 8%)  1 ( 8%) | 8 (36%)  6 (27%)  5 (23%)  3 (14%) | 6 (37%)  3 (19%)  2 (13%)  5 (31%) | 8 (67%)  0 ( 0%)  3 (25%)  1 ( 8%) | 7 (39%)  0 ( 0%)  2 (11%)  5 (28%) | 11 (48%)  4 (17%)  2 ( 9%)  6 (26%) | 23 (48%)  7 (15%)  5 (10%)  13 (27%) | 31 (48%)  11 (17%)  12 (19%)  10 (16%) |
| total | 6 (13%) | 7 (11%) | 12 (25%) | 22 (34%) |  | 16 (33%) | 12 (19%) | 14 (29%) | 23 (36%) | 64 (100%) |
| **Sleep quality ^2)^**  Poor sleep  Good sleep | 6 (100%)  0 ( 0%) | 4 (47%)  3 (43%) | 7 (58%)  5 (42%) | 12 (55%)  10 (45%) | 1 ( 8%)  11 (92%) | 9 (56%)  7 (44%) | 8 (35%)  15 (65%) | 4 (29%)  10 (71%) | 25 (39%)  39 (61%) | 26 (54%)  22 (46%) |
| Total | 6 (100%) | 7 (100%) | 12 (100%) | 22 (100 %) | 12 (100%) | 16 (100%) | 23 (100%) | 14 (100%) | 64 (100%) | 48 (100%) |
|  | *mean ± SE* | *mean ± SE* | *mean ± SE* | *mean ± SE* | *mean ± SE* | *mean ± SE* | *mean ± SE* | *mean ± SE* | *F df* | *F df* |
| **Age** | 25.50±3.38 | 26.31±2.38 | 31.50±3.06 | 29.20±2.50 | 44.00±3.40 | 32.06±3.02 | 44.14 ±1.49 | 37.11±3.16 | 7.81** 3,44 | 2.75 n.s. 3,60 |
| **Years living in Germany** | 19.33 ±4.04 | 18.54±2.10 | 23.17± 2.53 | 16.43±.44 | 22.88±2.89 | 13.63±2.75 | 15.29±2.52 | 7,76±1.64 | 1.84 n.s. 3,44 | 12.14** 3,60 |
| **Years of schooling** | 10.67± .56 | 11.46±.40 | 10.42±.65 | 10.71±.44 | 7.06±.72 | 7.69±1.18 | 5.14±.48 | 5.71±1.13 | 15.18** 3,44 | 5.13** 3,60 |

*f* = frequency, *SE* = standard error of means

1) statistics for life style and coping style (4 x 4 groups): Portuguese women: χ²= 8.31, *df* = 9, n.s.

Moroccan women: χ²= 10.20, *df* = 9, n.s.

1. statistics for life style and sleep quality (4 x 2 groups): Portuguese women: χ²= 8.88, *df* = 3, *p* < .05

Moroccan women: χ²= 8.11, *df* = 3, *p* < .05

statistics for dichotomized life style (German + rather German life style vs. rather traditional

+ traditional life style) and sleep quality (2 x 2 groups): Portuguese women: χ²= 3.80, *df* = 1, *p* = .05

Moroccan women: χ²= 5.78, *df* = 1, *p* = .05
