# Supplementary material for: Integration of immigrants into a new culture is related to poor sleep quality
Source: Health Qual Life Outcomes. 2008 Aug 10;6:61. doi: 10.1186/1477-7525-6-61 (PMC2518135; doi:10.1186/1477-7525-6-61)
Supplement: Additional file 2 — Descriptive statistics on coping style and sleep quality. This table shows the statistics for coping style and sleep quality in the Portuguese and Moroccan women interviewed. [file 1477-7525-6-61-S2.doc]

Table 2

Descriptive data for non-parametric χ²analyses on coping style in good and poor sleepers in samples of Portuguese (N = 48) and Moroccan immigrant women (N = 64)

|  | **Good sleep** | | **Poor sleep** | | **Total** | |
| --- | --- | --- | --- | --- | --- | --- |
|  | Portuguese  N % | Moroccan  N % | Portuguese  N % | Moroccan  N % | Portuguese  N % | Moroccan  N % |
| Totals | 26 54% | 39 61% | 22 46% | 25 39% | 48 100% | 64 100% |
| **Coping styles^1)^** |  |  |  |  |  |  |
| Monitors  Blunters  Adaptive C  unspecified | 9 39%  7 100%  2 40%  8 62% | 24 77%  4 36%  8 67%  3 30% | 14 61%  0 0%  3 60%  5 38% | 7 23%  7 64%  4 33%  7 70% | 23 48%  7 15%  5 10%  13 27% | 31 48%  11 17%  12 19%  10 16% |
|  | *mean ± SE* | *mean ± SE* | *mean ± SE* | *mean ± SE* | *mean ± SE* | *mean ± SE* |
| Age  BMI | 38.31±2.72  25.38± .88 | 30.79±1.94  20.46±.28 | 38.95±2.32  25.05± .73 | 33.16±2.41  21.24±.44 | 38.60±1.80  25.23±.58 | 31.72±1.51  20.77±.24 |
